# Decadal predictions of the North Atlantic CO_2_ uptake

**DOI:** 10.1038/ncomms11076

**Published:** 2016-03-30

**Authors:** Hongmei Li, Tatiana Ilyina, Wolfgang A. Müller, Frank Sienz

**Affiliations:** 1 Max Planck Institute for Meteorology, Bundesstraße 53, 20146 Hamburg, Germany

## Abstract

As a major CO_2_ sink, the North Atlantic, especially its subpolar gyre region, is essential for the global carbon cycle. Decadal fluctuations of CO_2_ uptake in the North Atlantic subpolar gyre region are associated with the evolution of the North Atlantic Oscillation, the Atlantic meridional overturning circulation, ocean mixing and sea surface temperature anomalies. While variations in the physical state of the ocean can be predicted several years in advance by initialization of Earth system models, predictability of CO_2_ uptake has remained unexplored. Here we investigate the predictability of CO_2_ uptake variations by initialization of the MPI-ESM decadal prediction system. We find large multi-year variability in oceanic CO_2_ uptake and demonstrate that its potential predictive skill in the western subpolar gyre region is up to 4–7 years. The predictive skill is mainly maintained in winter and is attributed to the improved physical state of the ocean.

## Abstract (web summary)

Predictability of variations in the ocean carbon sink has remained unexplored in previous decadal prediction studies based on modern Earth system models. Here, the authors show that potential predictive skill of the ocean CO_2_ uptake in the North Atlantic western subpolar gyre region is up to 4–7 years.

## Graphical abstract

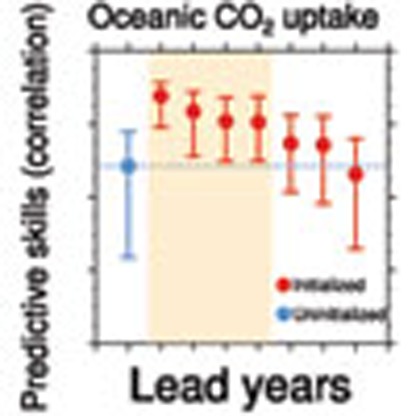

The world's oceans currently take over about 25–30% of anthropogenic CO_2_ from the atmosphere, and the North Atlantic is a key component of this oceanic carbon sink^1^,^2^. Steered by deep convection, most of the North Atlantic CO_2_ uptake takes place in the subpolar gyre (SPG) region, a region also characterized by pronounced variability in CO_2_ fluxes^3^,^4^,^5^. The variability of carbon uptake in the subpolar North Atlantic ocean was found to be largely related to variations of the North Atlantic Oscillation (NAO), the Atlantic meridional overturning circulation (AMOC), sea surface temperature (SST) and ocean-mixing strength, and all these processes are intrinsically related^3^,^5^,^6^,^7^,^8^,^9^. The SPG region experienced several abrupt warming and cooling events in the twentieth century, with SST increasing or decreasing by more than 1 °C in only a few years^10^,^11^,^12^,^13^. These changes are related to variations of the NAO and the associated AMOC, heat transports^11^,^14^, SPG circulation and Labrador Sea convection^15^,^16^. The key elements such as AMOC and SST have been shown to be predictable several years ahead by initialization of Earth system models (ESMs)^10^,^11^,^12^,^17^. However, no such investigation of predictability has been performed for the CO_2_ uptake so far. Are variations in the North Atlantic CO_2_ uptake represented in decadal prediction simulations with an ESM? And if so, to what extent is the CO_2_ uptake predictable?

We address these questions by using the Max Planck Institute-ESI (MPI-ESM) decadal prediction system^18^, which includes an assimilation simulation by nudging observations into the atmospheric and oceanic components of the ESM (see the Methods section) and a set of initialized retrospective prediction simulations (that is, starting from the assimilation run). For comparison, a set of uninitialized simulations is performed without any observations nudged. No carbon cycle observations are used in the assimilation run, so that the ocean biogeochemical processes perform as a passive component. Such an assimilation run, including both anthropogenic trends and natural fluctuations, allows us to investigate the spatial and temporal variability of the oceanic carbon uptake and its connection to local and large-scale physical processes. The initialized retrospective prediction simulations enable us to assess the predictability of carbon uptake. Here we mainly focus on interannual and longer timescale and if not stated otherwise use multi-year and annual mean data set. In this study, we focus on the SPG region because first it plays a prominent role in the oceanic carbon budget. Second, both the physical and the biogeochemical states of the ocean in the SPG region exhibit pronounced natural variability, which is governed by processes acting on decadal scales. Third, this region demonstrates robust predictive skill for the physical fields offering promise for predictions of the oceanic carbon uptake. Our analysis indicates that the North Atlantic CO_2_ uptake shows large spatial and temporal variations on decadal scale. Furthermore, these variations are better represented in the initialized simulations than in the uninitialized simulations. Direct comparison to observations indicates improved representation of the ocean surface pCO_2_ by initialization. We find the potential prediction skill of the western SPG CO_2_ uptake until lead time of 4–7 years. This predictability of CO_2_ uptake is related to the initialization of SST and AMOC by observations.

## Results

### Spatial variations of CO_2_ flux in the North Atlantic

**Figure 1:**
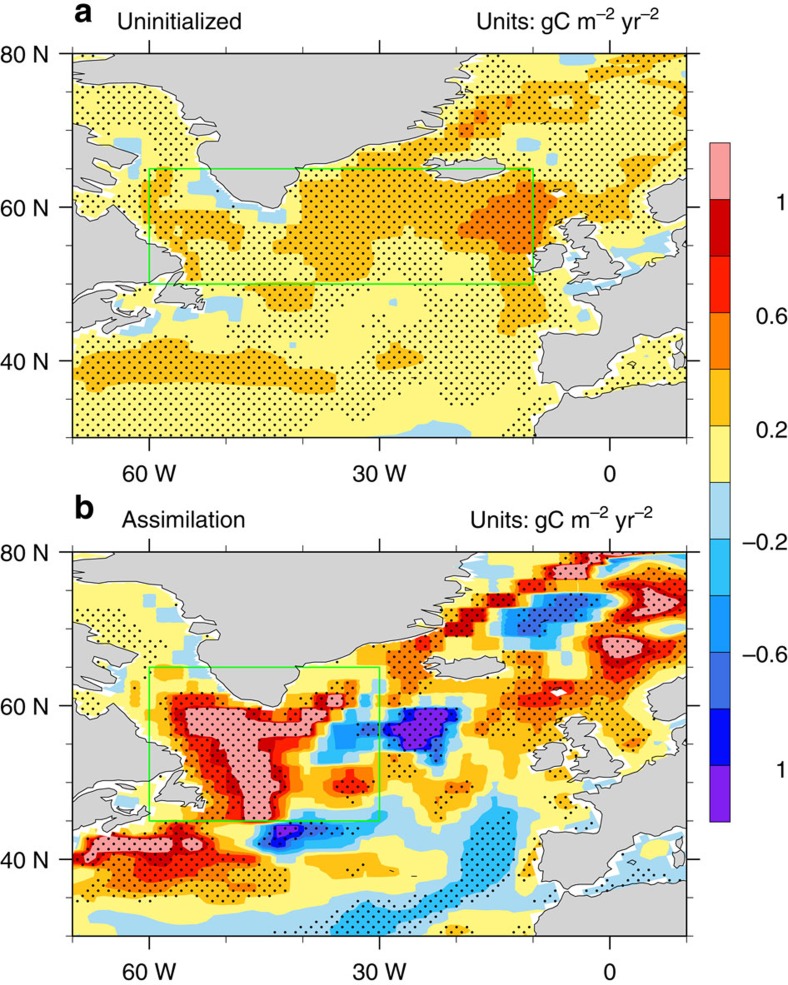
Trends of annual mean CO_2_ flux into the ocean from 1970 to 1995. Ensemble mean of uninitialized simulations (**a**) and assimilation simulation (**b**) (units: gC m^−2^ per year^2^). The trends are calculated based on linear regression. The dots show grid points where the trends are significant at 95% level, based on a two-sided *t*-test. The green box in **a** denotes the SPG region where the mid-1990s abrupt warming occurred^11^, and the green box in **b** denotes the western SPG region where the CO_2_ flux increases the most.

Spatial distributions of trends in CO_2_ flux during the 25 years before the mid-1990s SPG abrupt warming (that is, from 1970 to 1995) show a remarkably different pattern between the uninitialized and assimilation runs (Fig. 1). The oceanic CO_2_ uptake in the uninitialized simulations increases at about 0.2 gC m^−2^ per year^2^, following the increase in atmospheric CO_2_ due to rising fossil fuel carbon emissions, with a somewhat larger increase in the eastern part of the North Atlantic. The CO_2_ flux in the assimilation run does not increase uniformly in the North Atlantic; the largest increase is found in the western SPG region (Fig. 1b), and decreasing trends are found in the eastern SPG region. A zonal dipole distribution in delta pCO_2_ anomalies was also found in a previous study based on the simulations with an ocean only model^6^. This dipole trend pattern of CO_2_ uptake can be attributed to the NAO-related western–eastern heat loss gradient^19^ and the related ocean-mixing strength changes (Supplementary Fig. 1 and Supplementary Note 1). Regarding the mid-1990s warming, further decomposition of the SPG region by a previous study^20^ suggests a longer predictive skill in the western SPG region, which involves AMOC and meridional heat transport changes in response to persistent positive NAO phase from 1988 to 1995, and a shorter predictive skill in the eastern SPG region, which involves gyre circulation adjustment in response to NAO phase switch from positive to negative in 1995. Here we focus on the western SPG region with the largest trend of CO_2_ uptake and with longer prediction skill of the ocean physical state.

### Temporal evolution of CO_2_ uptake and related processes

**Figure 2:**
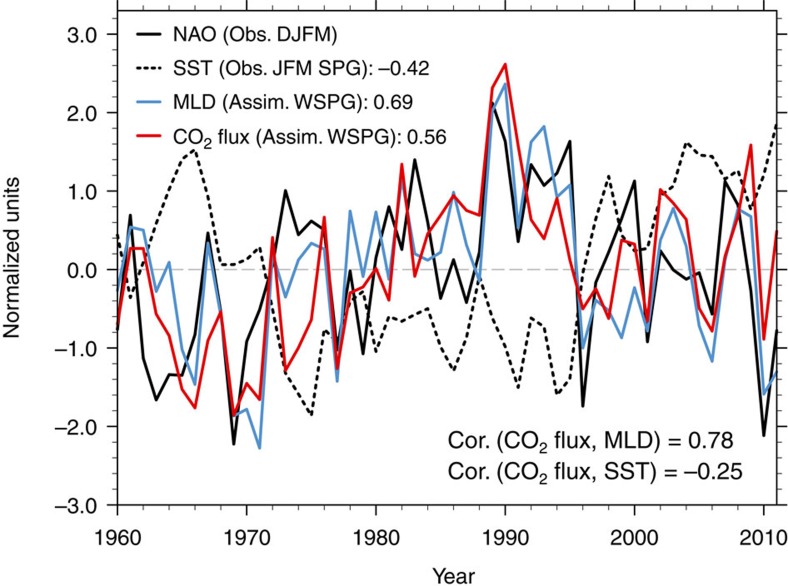
Normalized time series of CO_2_ flux and related physical variables. The observed (Obs.) DJFM NAO^21^, observed JFM SST^22^ in SPG region, western SPG (WSPG) MLD and CO_2_ flux calculated with the assimilation run are shown with black solid line, black dashed line, blue solid line and red solid line, respectively. The location of SPG and WSPG refers to green box in Fig. 1a,b, respectively. The correlation (Cor.) coefficients between time series and NAO (CO_2_ flux) are shown on the top (bottom) of the figure. The normalized time series are calculated by dividing individual variable anomalies with their respective standard deviation.

To further explore the temporal variability of the oceanic carbon uptake and how it is related to prominent local hydrodynamic processes in the SPG region, we compare the CO_2_ flux calculated in the assimilation run with the observed NAO index^21^, observed SST^22^ and mixed layer depth (MLD) in the assimilation run (Fig. 2). The western SPG MLD and CO_2_ flux from our model results are highly correlated (with a correlation coefficient of 0.78), and both are highly correlated (with correlation coefficients of 0.69 and 0.56, respectively) with the observed December–January–February–March (DJFM) NAO index. The correlations are significantly different from zero with *P* values below 0.01. Consistent with previous model studies^6^, the close connection between the NAO and the North Atlantic CO_2_ uptake at interannual and decadal timescales is also reproduced in the assimilation run. On the one hand, the heat loss related to a positive NAO leads to an enhancement of the ocean mixing and deep-water formation in the North Atlantic^19^; this is indicated by the relatively high correlation (0.69) between NAO and MLD. Moreover, the SST anomalies in the SPG region with deep convection recur from winter to winter through a re-emergence process^23^,^24^, which works as follows. The winter thermal anomalies related to positive NAO remain at depth below the shallow summer mixed layer, these anomalies persist through summer and are partially re-entrained into the following winter mixed layer. On the other hand, the North Atlantic atmospheric circulation changes can also affect CO_2_ uptake through the associated changes in AMOC and the corresponding meridional heat transport changes. Enhanced northward heat transport leads to increased SST thereby affecting the oceanic carbon uptake in the SPG. These processes lag the NAO changes by several years^14^. Accordingly, given all the processes, the correlation between CO_2_ flux and SST is not significant in our simulations.

### Potential predictive skill of CO_2_ flux and SST

The state-of-the-art decadal prediction systems consistently identify the North Atlantic as a key region with pronounced forecast skill for different parameters of the climate system^25^,^26^,^27^,^28^,^29^,^30^,^31^. Several abrupt climate events in the twentieth century have been shown to be predictable several years ahead^10^,^11^,^12^,^13^. The SPG abrupt warming and cooling events are also well captured in our initialized simulations, whereas the uninitialized simulations only capture the long-term warming trend and display large spread among ensemble members (Supplementary Fig. 2 and Supplementary Note 2). Given the robust predictive skill of the physical state of the ocean, can variations in the North Atlantic CO_2_ uptake be predicted as well? A previous study explored multi-year predictability of tropical marine productivity and found a predictive skill of 3 years, whereas the predictive skill of SST there is only 1 year^32^. In our analysis, as indicated by the initialized time series at different lead times (Supplementary Fig. 3 and Supplementary Note 3), the variability of CO_2_ flux, which is absent in the uninitialized simulations, can be reproduced by the model with initialization several years in advance and implies predictability of CO_2_ uptake.

**Figure 3:**
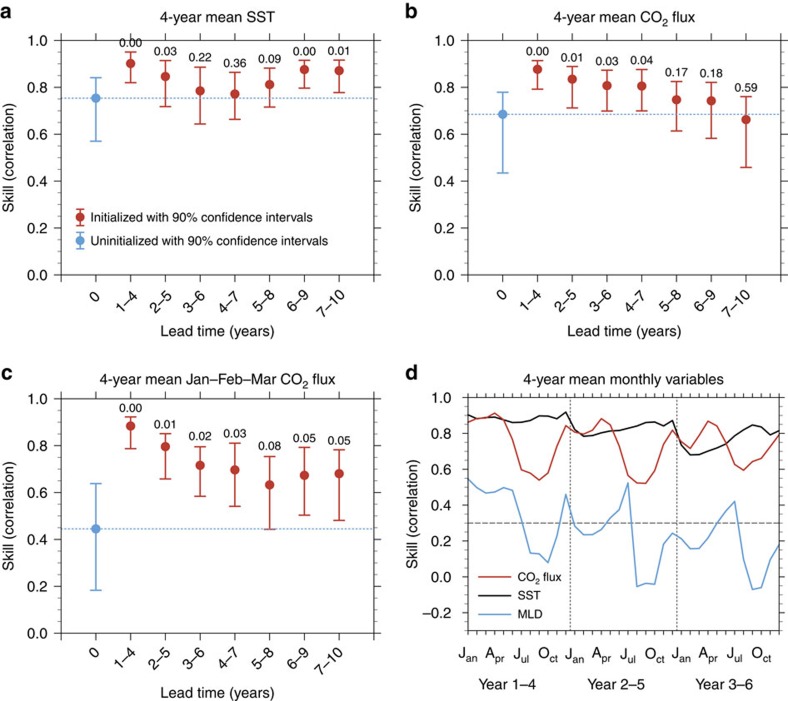
Potential predictive skill of CO_2_ flux and related physical variables. Correlation skill of ensemble mean of 4-year mean SST (**a**), 4-year mean CO_2_ flux into the ocean (**b**), seasonally stratified 4-year mean CO_2_ flux into the ocean (late winter, that is, January–February–March) (**c**) and monthly stratified 4-year mean CO_2_ flux, SST and MLD (**d**) in the western SPG region. Shown are uninitialized (blue dot in **a**–**c**) and initialized (red dots in **a**–**c**) simulations at different lead time verified against assimilation. The correlations are calculated from 4-year mean predictions (that is, years 1–4, 2–5 and so on) of initialized simulations, and 4-year running mean of the corresponding assimilation and uninitialized time series. To ensure that the number of validation years is the same for both initialized and uninitialized simulations, we use the common time period from 1967–1970 mean to 2008–2011 mean. The blue dashed line in **a**–**c** extends the uninitialized correlation for easy comparison. The vertical lines in **a**–**c** provide 90% confidence intervals based on a bootstrap approach^46^. The numbers on the top of the bars in **a**–**c** show the *P* values based on the hypothesis that the difference of correlations between the initialized and uninitialized simulations is smaller or equal to zero based on 1,000 bootstrapped resamples. The potential predictive skills of monthly stratified 4-year mean CO_2_ flux, SST and MLD in **d** are shown with red, black and blue curves, respectively. Three-month running mean is applied before monthly correlation estimation in **d**. The horizontal dashed grey line in **d** shows the 95% level of significance based on a two-sided *t*-test. The vertical dashed grey lines in **d** are added to better distinguish different lead year results.

Owing to lack of observational data, we first use model fields calculated in the assimilation run as a proxy to quantify the potential predictive skill of SST and of the CO_2_ flux into the ocean (Fig. 3, see the Methods section). The correlation coefficients of the initialized 4-year mean SST exceed the uninitialized correlations for lead times of 1–4 and 2–5 years. The correlation goes down in the intermediate lead time, however, it recovers from a lead time of 5–8 years. The results suggest improved potential predictive skills of the background ocean physical fields. The correlation coefficient of initialized CO_2_ flux is significantly higher than the uninitialized correlation until a lead time of 4–7 years. The *P* values are lower than 0.05 until the lead time of 4–7 years suggesting significant improvement of potential predictive skill due to initialization of the physical states constrained by observations.

Further investigation of the potential predictive skill with seasonal and monthly time series (Fig. 3c,d) reveals that the high predictive skill of CO_2_ flux in the initialized simulations is mainly maintained during winter months. This is related to the seasonal shift of processes in regulating the CO_2_ uptake and the fact of assimilation for the decadal prediction system. In winter, the CO_2_ uptake in western SPG region is regulated primarily by physical process such as the ocean-mixing strength and ocean circulation^33^,^34^,^35^. The variations of ocean physical fields are well represented in the initialized simulations, as the corresponding initial states are constrained by observations through assimilation. However, in spring and early summer, when the ocean warms, the biological primary production draws down seawater pCO_2_ and regulates the oceanic CO_2_ uptake in the western SPG region^33^,^34^,^35^; the correlation of the initialized simulations goes down from May, as no ocean biological observations are assimilated into the system.

We further explore possible causes of the potential predictive skill of CO_2_ uptake in comparison with that of the SST and MLD (Fig. 3d). The SST correlations peak in December and go down from late winter, which is coherent with that of the MLD and the CO_2_ uptake. It suggests that both the evolution of the ocean thermal state and the local mixing strength contribute to the predictive skill of CO_2_ uptake in winter. Although the correlation skill of MLD is generally lower than that of CO_2_ uptake, both share similar seasonal cycle of predictive skill, indicating effects of MLD changes in maintaining the predictive skill of CO_2_ uptake. Moreover, the potential predictive skill of CO_2_ uptake is related to the AMOC variability. As revealed by previous studies, the predictive skill of SPG SST is assured by initialization of the AMOC variability^27^, and the AMOC shows predictive skill up to 4 years^17^. The potential predictive skill of AMOC in our system is up to 2–5 years (figure omitted). The AMOC at lead time of 1 year is highly correlated with CO_2_ flux at lead time of 1 year and onwards (Supplementary Fig. 4 and Supplementary Note 4). An observational study also suggested a close connection between the AMOC and the CO_2_ uptake in SPG region^3^.

From these our results suggest that the potential predictive skill of CO_2_ uptake in the western SPG is up to 4–7 years. The predictive skill is mainly attributed to the combination of improved ocean physical states and circulation variability, primarily in winter.

### Evaluation of model predictions against observations

**Figure 4:**
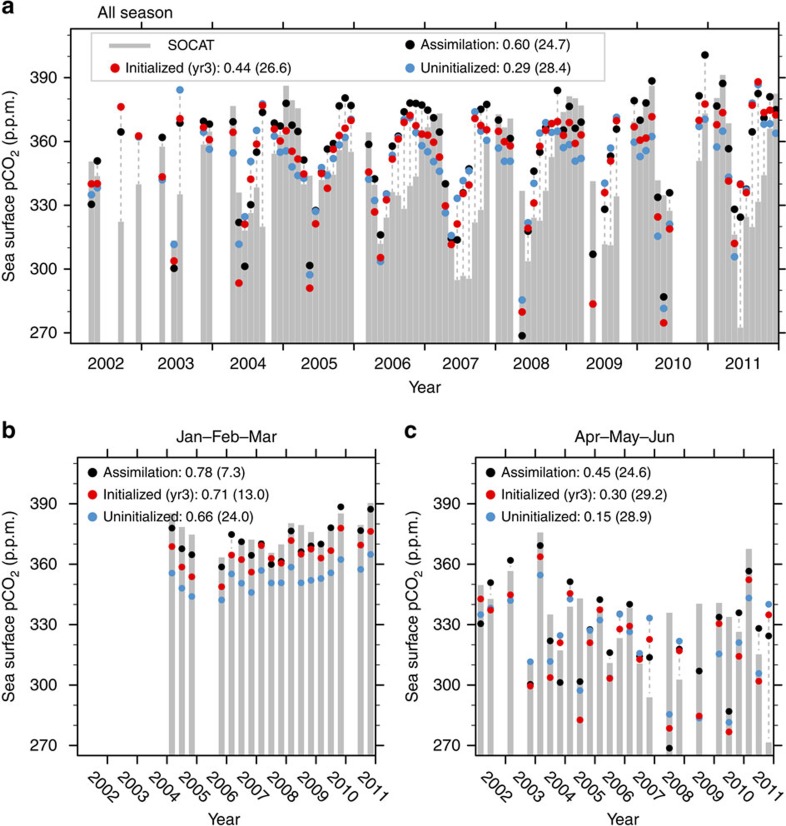
Observed and model simulated monthly mean ocean surface pCO_2_ in western SPG region. (**a**) All season time series, (**b**) only January–February–March are shown and (**c**) only April–May–June are shown. The SOCAT observations^36^ are shown with grey bars, and model output of assimilation, uninitialized simulations and initialized simulations at a lead time of 3 years (yr3) are shown in black, blue and red dots, respectively. The grey dashed lines connect outlier dots with the grey bars to make the comparison clearer. The numbers in legend are correlation coefficients and root mean squared error (in brackets) between model simulations and SOCAT observations. For the all season time series in **a** the correlations of the assimilation and initialized simulations at lead time of 3 years are significantly larger than that of the uninitialized simulations at 95% significant level based on a bootstrap test. Note the SOCAT observational data are not continuously distributed in space and time; the model simulations are averaged over the grid points where SOCAT data are available.

The high potential predictability of CO_2_ uptake provides a basis for assessing our predictions against observations using the surface ocean CO_2_ atlas (SOCAT) measurement^36^. Although observational data from SOCAT are sparse in the SPG region, these are the best ocean surface observations we can get for this region. The ocean surface pCO_2_ in the SPG region peaks in winter as a result of the enhanced vertical supply of carbon from the intermediate waters by deep convection; surface pCO_2_ values reach a minimum in summer due to biological draw down^33^,^34^,^35^ (Fig. 4). The initialized predictions produce ocean surface pCO_2_ closer to SOCAT observation than the uninitialized simulations as indicated by the correlations and root mean squared errors. The correlations of assimilation (0.60) and initialized simulation at a lead time of 3 years (0.44) are significantly larger than the correlation of initialized simulation (0.29) at 95% significance level. The root mean squared error is lower in the assimilation and initialized simulations than in the uninitialized simulation. As we use monthly data due to lack of continuous observations, the better performances of initialized simulations are partially due to better representation of the seasonal cycle in the initialized simulations. We further separate the time series seasonally, and find that in addition to the seasonal cycle there is an improvement of the initialized run against the uninitialized run particularly in the winter months when the pCO_2_ is high. The root mean squared error of ocean surface pCO_2_ is much smaller in the assimilation (7.3 p.p.m.) and initialized simulations at a lead time of 3 years (13.0 p.p.m.) than in the uninitialized simulations (24.0 p.p.m.; Fig. 4b). The coherences between model simulations and observations are generally lower in spring months (Fig. 4c). The improvement of prediction in individual seasons further demonstrates that the interannual variations of oceanic carbon cycle are improved in the initialized simulations. The higher correlations between SOCAT and initialized simulations and the lower root mean squared error of the initialized simulations against SOCAT confirm that the oceanic carbon cycle can be predicted several years ahead by initialization of the ESM (Supplementary Fig. 5 and Supplementary Note 5). However, owing to temporal and spatial gaps in observations, the precise prediction skill with respect to observations cannot be estimated. Comparison of simulations against SOCAT observations only confirm that prediction of the state of the oceanic carbon cycle is improved by initialization of the physical ocean state with observations.

## Discussion

We have focused here on the impacts of the ocean physical state on the variability and predictability of the North Atlantic CO_2_ uptake. The drawdown of pCO_2_ by biological processes such as carbon fixation during seasonal phytoplankton growth, with consequent export of carbon into the deep ocean is also critical for the air–sea carbon exchange in the nutrient-rich SPG region^37^. These marine biogeochemical processes themselves are influenced by variability in climate and circulation. For instance, an increase in ocean temperature leads to a decrease of CO_2_ uptake through decrease in solubility. Biological production starts in spring when the ocean warms up. This draws down the ocean surface pCO_2_ and enhances CO_2_ uptake. However, the growth of phytoplankton in the North Atlantic is more limited by nutrient supply than by temperature. The nutrient supply, in turn, is constrained by the enhanced thermal stratification associated with the ocean warming, thus resulting in a decrease of primary production^38^. Therefore, while the biological CO_2_ drawdown regulates the seasonal cycle of CO_2_ flux, the physical regulation is more important for interannual and decadal variations of CO_2_ flux in the SPG region^39^. Our findings show that there is an improved representation of the oceanic carbon flux because the background oceanic circulation and thermal state are well reproduced. Physical processes such as the ocean thermal state, local mixing and large-scale circulation contribute to the predictability of CO_2_ uptake. It requires further large ensembles of multi-model decadal prediction simulations and sensitivity experiments to disentangle the relative role of an individual physical mechanism on the predictability of CO_2_ uptake.

We find that beside the trend due to CO_2_ emissions, predictions of the oceanic uptake and storage of carbon show considerable decadal variations that are not fully captured in the uninitialized simulations with modern ESMs. We demonstrate that variations in the oceanic carbon cycle can be predicted several years ahead. Hence, predictions of the North Atlantic carbon sink considering both anthropogenic change and natural fluctuations are important to understand the evolution of climate and ocean acidification and to reduce uncertainties in CO_2_ uptake estimates. Predictions of the oceanic carbon sink are also necessary to provide information to monitoring programs aimed at the present and future oceanic carbon sink.

## Methods

### Model description

We use the MPI-ESM^40^ to conduct historical (uninitialized) experiments and retrospective decadal prediction (initialized) experiments for the period 1961–2013. A low-resolution configuration (MPI-ESM-LR) is used with the resolution of the ocean model being 1.5° on average with 40 vertical levels. The ocean component of MPI-ESM-LR use bipolar configuration, with one pole over Greenland and another over Antarctica, so the resolution in the North Atlantic SPG region is from ∼20 km in the north (65° N) to ∼70 km in the south (45° N). MPI-ESM-LR is capable of producing North Atlantic deep convection confined to the Labrador Sea, which is close to observation^41^. The ocean biogeochemistry component of MPI-ESM is represented by the Hamburg Ocean Carbon Cycle Model (HAMOCC)^42^.

### Numerical simulations

An ensemble of 10 uninitialized historical simulations and RCP4.5 scenario simulations is performed for the periods 1850–2005 and 2006–2013, respectively. The uninitialized experiments are started from a preindustrial control simulation and forced with historical greenhouse gas and aerosol concentrations together with solar variability and volcanic eruptions. The initial conditions of the uninitialized run ensemble members differ based on the preindustrial control simulation starting from every 50 years. On the basis of an assimilation run, an ensemble of 10 initialized simulations is started on 1 January in every year over the period 1960–2012. The ensemble members of the initialized simulations are generated with lagged 1-day initialization, that is, the runs start from 10 consecutive days centred on 1 January. Each initialized simulation has a length of 10 years^18^. The assimilation experiment is also performed with MPI-ESM-LR. For the oceanic component, monthly temperature and salinity anomalies from the ECMWF ocean reanalysis system 4 (ORAS4)^43^ are added to the model climatology and nudged into the model state at every time step. For the atmospheric component, globally full-field temperature, vorticity, divergence and surface pressure data from the ECMWF ERA40^44^ and ERA-Interim^45^ reanalyses are nudged.

### Predictive skill assessment

We mainly show ensemble mean results in this study. For the uninitialized experiments, the anomalies are calculated by removing the climatology of the ensemble mean. For the initialized experiments, the anomalies are calculated in terms of the climatology of the ensemble member and the lead time. The observed temporal evolution of the DJFM NAO index (station based)^21^ and the SST^22^ in SPG is investigated together with the mixing and CO_2_ flux around the SPG region in the assimilation run. We use the fields in the assimilation run as a proxy to quantify the historical variability and to estimate the potential predictive skill of CO_2_ uptake. The potential predictions are verified with skill scores based on anomaly correlation coefficients against the assimilation run. As short-term/small-scale variability may add noise and hence reduce predictive skill, temporal and spatial averaging was recommended^46^ and has been used in most decadal prediction studies^25^,^31^,^47^. A set of 4-year mean (that is, 1–4, 2–5, …, 7–10 years) predictions is verified in this study against corresponding running mean of the assimilation and uninitialized simulations.

### Bootstrap approach for significance test

Statistical tests and confidence intervals of the correlations in Figs 3 and 4 are calculated through a bootstrapping approach^46^. The resampling scheme considers uncertainty arising from both ensemble size and hindcast period. A block size of 2 is used to account for autocorrelation in the SOCAT time series (Fig. 4). Note that the significance of correlation differences in Fig. 3 remains unaltered for the same block size.

### Conversion of ocean surface CO_2_ observation data

The gridded observations of the SOCAT^36^ surface ocean fugacity of CO_2_ (fCO_2_) from 2002 to 2011 are used to verify model simulations. The fugacity is converted to partial pressure^48^. As observational data are not continuously distributed in space and time, for the model evaluation presented in Fig. 4 and Supplementary Fig. 5 we only consider those model grid points where SOCAT data are available.

### Code availability

The MPI-ESM model is freely available to the scientific community. The code can be accessed with a license agreement on the Max Planck Institute for Meteorology model distribution website: http://www.mpimet.mpg.de/en/science/models/license/

## Additional information

**How to cite this article:** Li, H. *et al.* Decadal predictions of the North Atlantic CO_2_ uptake. *Nat. Commun.* 7:11076 doi: 10.1038/ncomms11076 (2016).

## Supplementary Material

Supplementary Information Supplementary Figures 1-5, Supplementary Notes 1-5 and Supplementary References.
