# Supplementary material for: Decadal predictions of the North Atlantic CO2 uptake
Source: Nat Commun. 2016 Mar 30;7:11076. doi: 10.1038/ncomms11076 (PMC4820896; doi:10.1038/ncomms11076)
Supplement: Supplementary Information — Supplementary Figures 1-5, Supplementary Notes 1-5 and Supplementary References. [file ncomms11076-s1.pdf]

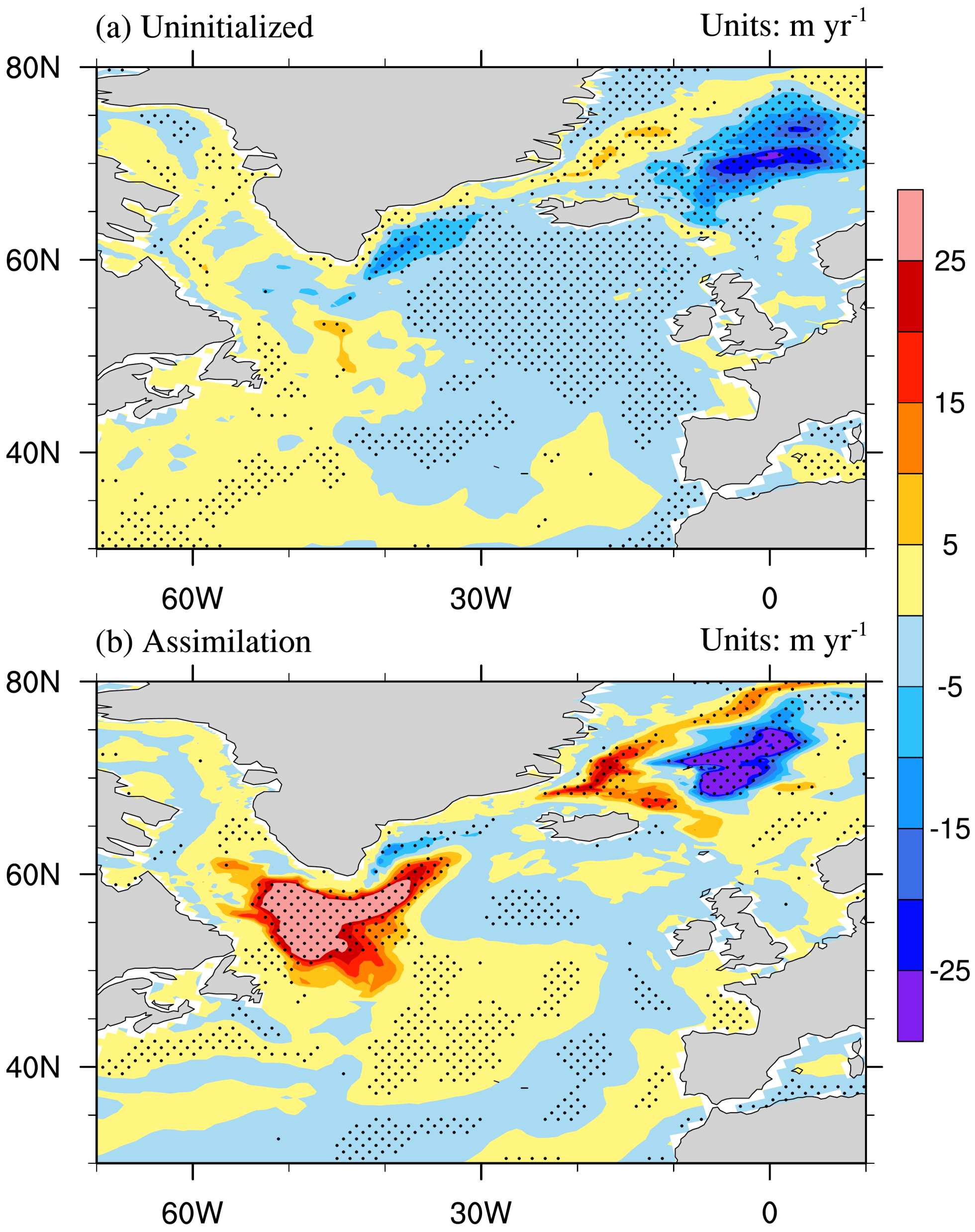

Supplementary Figure 1 | Trends of annual mean maximum ocean mixed layer depth. Trends from uninitialized simulations (a) and assimilation simulation (b) from 1970-1995 (units: m yr^{-1}). The dots show grids where the trends are significant at 95% level based on a 2-sided t-test.

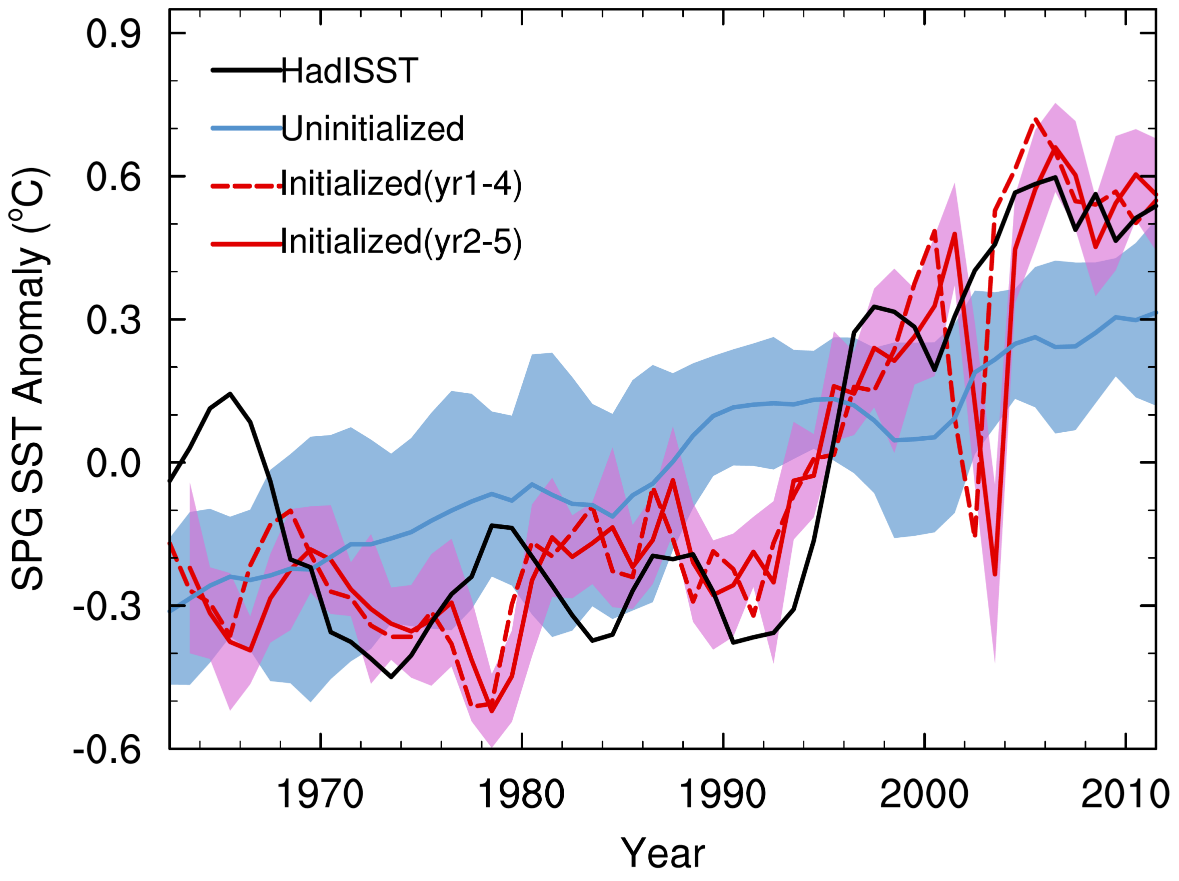

Supplementary Figure 2 | Time series of 4 year mean SST anomaly in the SPG region. Shown are 4-year running mean SST anomaly in the SPG region (see green box in Fig. 1a) from HadISST[1] (black curve), uninitialized simulations (blue curve), initialized simulations at lead times of 1-4 years (dashed red curve) and of 2-5 years (solid red curve). The standard deviation of uninitialized simulations and initialized simulations at lead time of 2-5 years are shown with blue and red shadings.

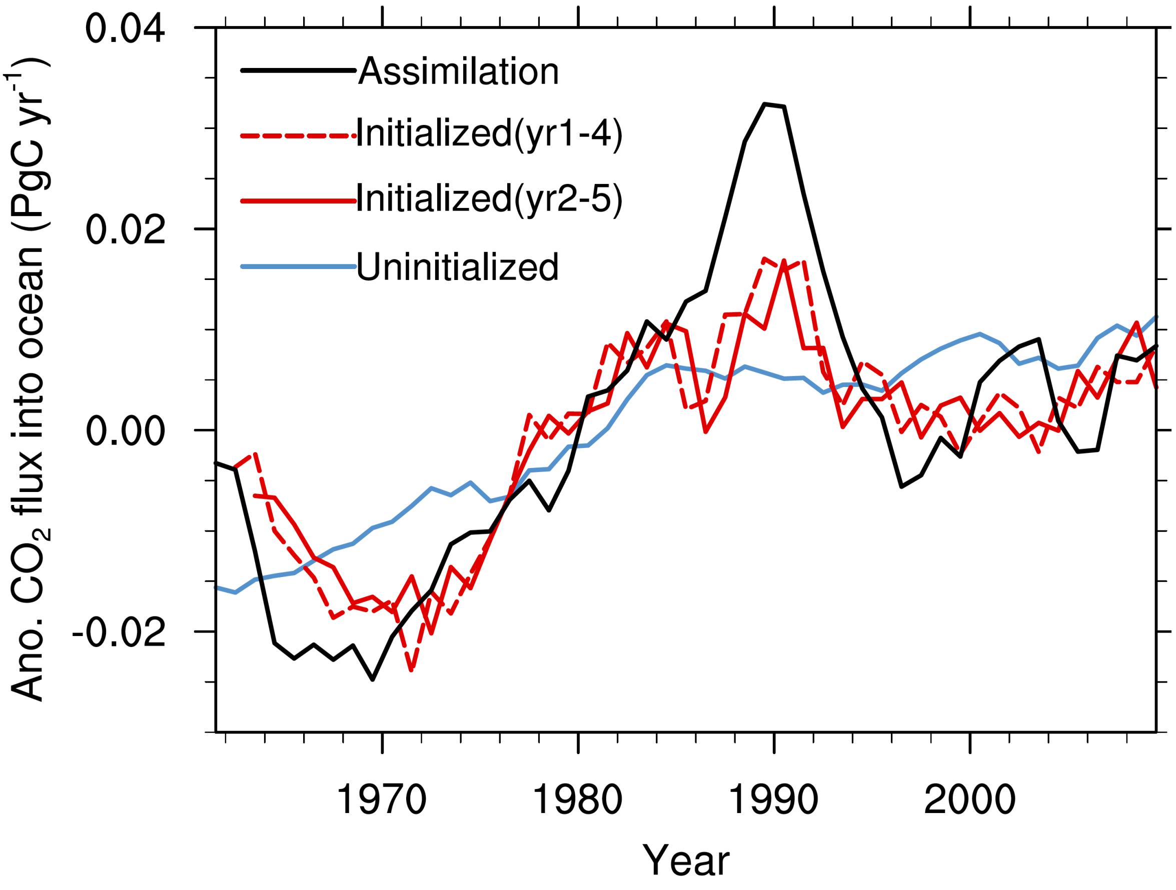

Supplementary Figure 3 | Temporal evolution of 4 year mean anomalous CO_2 flux into the ocean in the western SPG region. The western SPG region refers to green box in Fig. 1b, i.e., 60°-30°W, 45°-65°N. The results from uninitialized simulations, assimilation simulations, and initialized simulations at lead time of 1-4 years and 2-5 years are shown as blue solid line, black solid line, red dashed line, and red solid line.

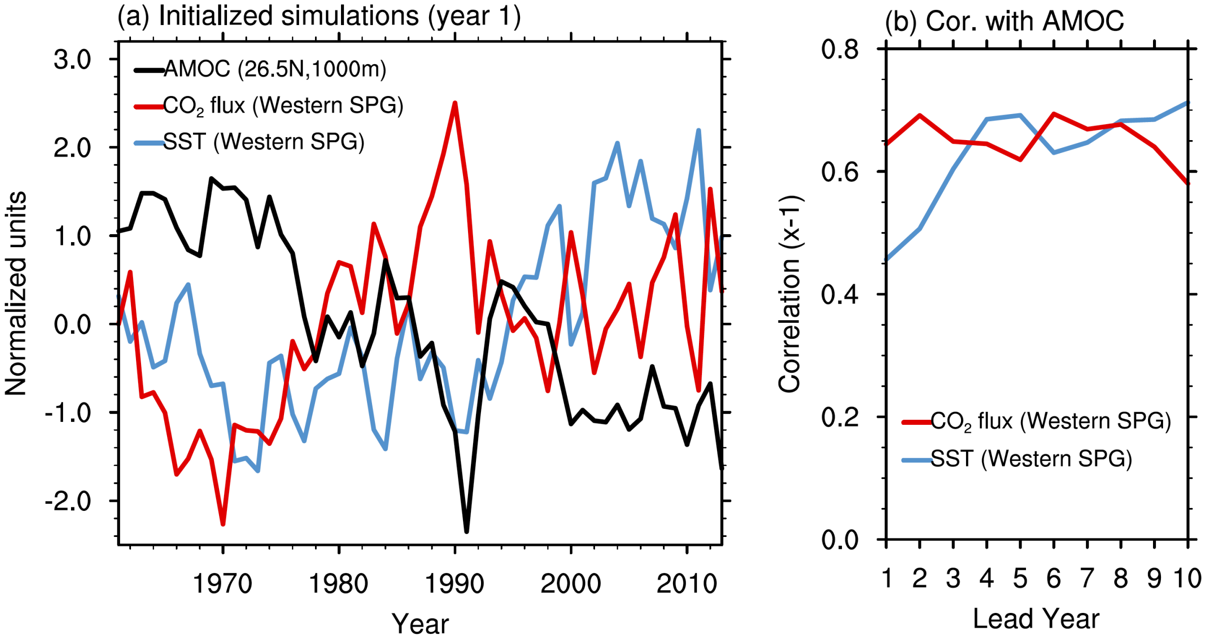

Supplementary Figure 4 | Temporal evolution of AMOC, CO_2 flux, SST, and lag correlations among them. (a) Temporal evolution of AMOC at 26.5°N and 1000m depth (black curve), CO_2 flux into the ocean (red curve) and SST (blue curve) in the western SPG region from initialized simulations at lead time of 1 year. (b) Correlations between the AMOC and CO_2 flux (red curve), and SST (blue curve) in the western SPG region, the lead time for AMOC is fixed to 1 year, and lead time for CO_2 flux and SST varies from 1-10 years as the X-Axis shows. Note that the correlations are multiplied by -1. The normalized time series are calculated by dividing individual variable anomalies with their respective standard deviation.

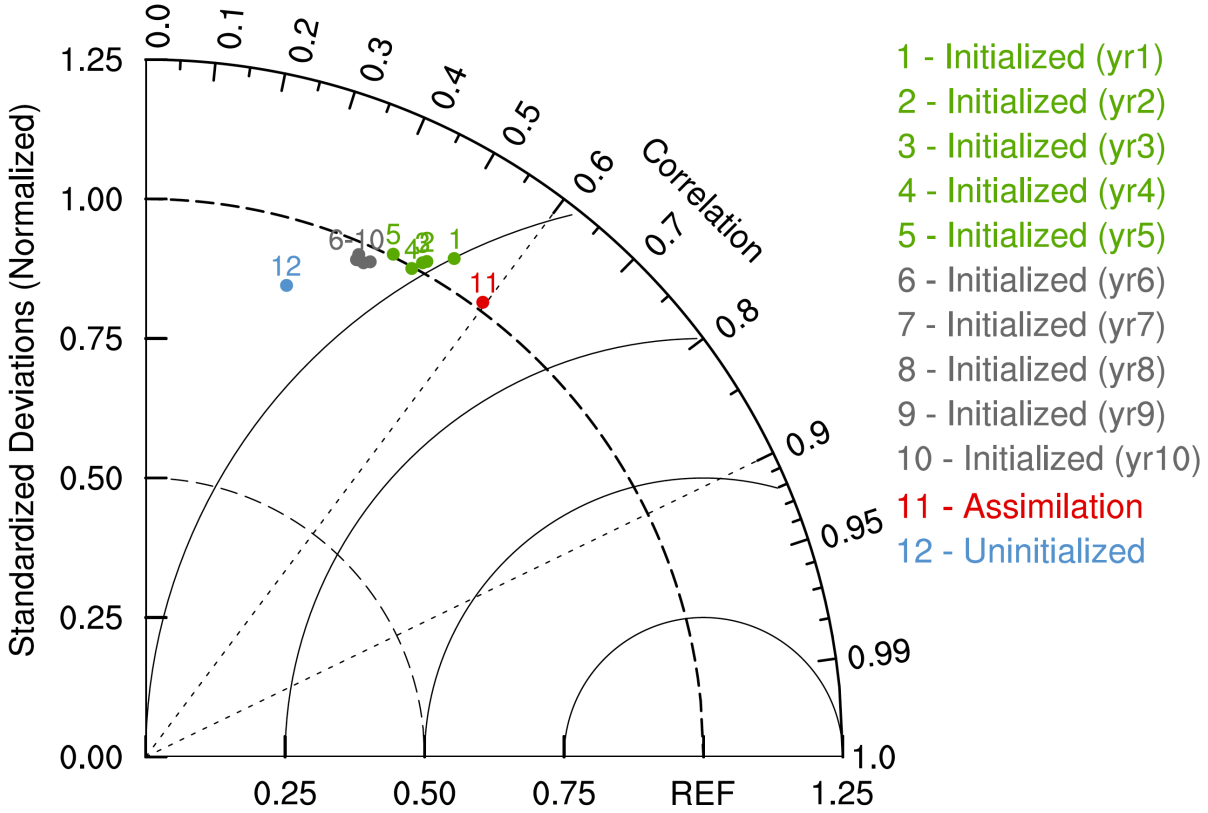

Supplementary Figure 5 | Taylor diagram on western SPG ocean surface pCO_2. The correlation between model simulation and SOCAT observation[2] is given by the azimuthal position, the radial distance from the origin is the ratio of the normalized standard deviation[3]. REF refers to SOCAT, the root-mean-square-error is shown by the distance from the REF on the X-Axis. The results from uninitialized runs, assimilation run, initialized runs from lead time of 1-5 years, and initialized runs from lead time of 6-10 years are shown in blue, red, green, and grey, respectively.

Supplementary Note 1: Reconstruction of trends in oceanic mixing strength

The CO_2 uptake in the assimilation run shows an increasing trend in the western SPG region and a decreasing trend in the eastern SPG region (Fig. 1b); this zonal dipole trend pattern can be attributed to the NAO-related western-eastern heat loss gradient. This mechanism operates as follows. Large ocean heat loss related to a positive NAO enhances the convection activity in the western SPG region (the Labrador Sea)[4]. Hence, the mixing there is enhanced, leading to more CO_2 uptake. Meanwhile, the heat loss in the eastern SPG is much smaller than in the western SPG, the dense and saline water intrusion along the isopycnal from the west to the east weakens the mixing in the eastern SPG, thereby leading to less CO_2 uptake. Here we estimate trends of ocean mixed layer depth from 1970-1995 (Supplementary Fig. 1). The assimilation simulation produces reverse mixing trends in the western SPG and the eastern SPG, however, the uninitialized simulations show uniform negative trend of mixing strength in the whole SPG region.

Supplementary Note 2: Predictability of SST

Before investigating the predictive skill of the oceanic carbon cycle, we estimate the predictability of ocean physical field such as SST as shown in Supplementary Fig. 2. The abrupt warming in the 1990s[5] in SPG region is well captured in the MPI-ESM-LR initialized simulations, the spread among ensemble members represented by standard deviation is relatively small. However, the uninitialized simulations mainly show a long term warming trend, and the spread among ensemble members of uninitialized simulations is much larger than that of the initialized simulations.

Supplementary Note 3: Predictability of CO_2 uptake

The temporal evolution of CO_2 uptake in the western SPG from the uninitialized simulations, assimilation simulation, and initialized simulations at lead times of 1-4 years and 2-5 years are shown in Supplementary Fig. 3. The CO_2 uptake in the uninitialized simulations shows mainly an increasing trend following the increase of anthropogenic CO_2 emissions. However, both the assimilation and the initialized simulations show considerable variations on decadal scale in addition to a long-term trend; the CO_2 uptake decreases in 1960s, increases during 1970s-1980s, and it decreases again in 1990s. The decline of CO_2 uptake in the 1990s is

consistent with observational results, a rapid decline of CO_2 uptake in the subpolar North Atlantic Ocean between 1990 and 2006 was documented[6]. Moreover, the variability of CO_2 uptake, which is missing in the uninitialized simulations, can be reproduced by MPI-ESM-LR with initialization several years in advance.

Supplementary Note 4: Attribution of high predictive skill of CO_2 uptake

As shown in Fig. 3, the variations of SST and CO_2 uptake in the western SPG region are connected to each other, and their predictability may be maintained by common physical mechanism. The initialization of Atlantic meridional overturning circulation (AMOC) variability contributes to the predictive skill of SST in the SPG region[7]. It is also true for this experiment, the correlations between AMOC at 26.5°N at lead time of 1 year and SST in western SPG region at lead time from 3 years onwards are higher than 0.6 (Supplementary Fig. 4b). The predictive skill of CO_2 uptake in the western SPG region is also assured by initialization of the AMOC. The AMOC is highly correlated with the western SPG CO_2 flux simultaneously (Supplementary Fig. 4a), and the correlations between AMOC and western SPG CO_2 flux are higher than 0.6 starting from lead time of 1 year till lead time of 9 years (Supplementary Fig. 4b). To date the trend of AMOC in the past is unclear due to lack of observations. The long-term decreasing trend of AMOC in the initialized simulations may be due to the negative trend of the assimilated data from ORA-S4 which also exhibits a negative trend[8].

Supplementary Note 5: Predictive skill of ocean surface pCO_2

Here we summarize the correlations, variability ratios, and root-mean-square-errors of model simulated ocean surface pCO_2 in the western SPG region against SOCAT observations[2] in a Taylor diagram[3] (Supplementary Fig. 5). The correlations between SOCAT and initialized simulations at a lead time of 10 years is even larger than the correlations between SOCAT and uninitialized simulations. The root-mean-square-error against SOCAT observation are generally lower in the initialized simulations than in the uninitialized simulations. It indicates that the predictive skill of oceanic carbon cycle is improved by initializing the physical fields of the earth system model. As we use monthly mean data, the improvement may be partially but not dominantly contributed by the seasonal cycle, which is in some extent interrupted by the missing measurement and the location change of the measurements.

Supplementary References

1 Rayner, N. A. *et al.* Global analyses of sea surface temperature, sea ice, and night marine air temperature since the late nineteenth century. *Journal of Geophysical Research-Atmospheres* **108**, doi:10.1029/2002jd002670 (2003).

2 Bakker, D. C. E. *et al.* An update to the Surface Ocean CO2 Atlas (SOCAT version 2). *Earth Syst. Sci. Data* **6**, 69-90 (2014).

3 Taylor, K. E. Summarizing multiple aspects of model performance in a single diagram. *J. Geophys. Res.* **106**, 7183-7192, doi:10.1029/2000JD900719 (2001).

4 Eden, C. & Willebrand, J. Mechanism of Interannual to Decadal Variability of the North Atlantic Circulation. *J. Climate* **14**, 2266–2280 (2001).

5 Robson, J. I., Sutton, R. T. & Smith, D. M. Initialized decadal predictions of the rapid warming of the North Atlantic Ocean in the mid 1990s. *Geophysical Research Letters* **39**, doi:10.1029/2012gl053370 (2012).

6 Pérez, F. F. *et al.* Atlantic Ocean CO2 uptake reduced by weakening of the meridional overturning circulation. *Nature Geoscience* **6**, 146-152, doi:10.1038/ngeo1680 (2013).

7 Matei, D. *et al.* Two Tales of Initializing Decadal Climate Prediction Experiments with the ECHAM5/MPI-OM Model. *Journal of Climate* **25**, 8502-8523, doi:10.1175/jcli-d-11-00633.1 (2012).

8 Balmaseda, M. A., Mogensen, K. & Weaver, A. T. Evaluation of the ECMWF ocean reanalysis system ORAS4. *Quarterly Journal of the Royal Meteorological Society* **139**, 12-41, doi:10.1002/qj.2063 (2013).
